# High mutations in fatty acid metabolism contribute to a better prognosis of small‐cell lung cancer patients treated with chemotherapy

**DOI:** 10.1002/cam4.4290

**Published:** 2021-09-26

**Authors:** Qiong Lyu, Weiliang Zhu, Ting Wei, Weimin Ding, Manming Cao, Qiongyao Wang, Linlang Guo, Peng Luo, Jian Zhang

**Affiliations:** ^1^ Department of Oncology Zhujiang Hospital Southern Medical University Guangzhou China; ^2^ Department of Pathology Zhujiang Hospital Southern Medical University Guangzhou China

**Keywords:** biomarker, fatty acid metabolic pathway, prognosis, small‐cell lung cancer (SCLC), whole exome sequencing (WES)

## Abstract

**Background:**

The majority of patients with small‐cell lung cancer (SCLC) show a good response in the early stages of treatment, but more than 90% of patients will develop drug resistance. Therefore, biomarkers are urgently needed to identify patients who can benefit from systemic treatment.

**Methods:**

We prospectively enrolled 52 extensive‐stage SCLC patients before treatment from a local hospital to identify mutations related to patient prognosis, and verified them in the published Jiang's cohort and George's cohort.

**Results:**

We found that patients with high mutations (mut‐high) in the fatty acid (FA) metabolism pathway had a longer progression‐free survival (PFS) in the local hospital cohort (HR = 0.446, 95% CI, 0.207–0.959, p = 0.0387) and a longer overall survival (OS) in Jiang's cohort (HR = 0.549, 95% CI, 0.314–0.960, p = 0.0351) than patients with low mutations (mut‐low). Multivariate analysis suggested that mut‐high status was an independent prognostic factor in both cohorts. George's cohort verified that mut‐high status was associated with a longer OS than mut‐low status (HR = 0.730, 95% CI 0.440–1.220, p = 0.2277). The possible mechanisms were as follows: the frequency of mutated FA synthase (FASN) in the mut‐high group was greater than that in the mut‐low group, and pathways related to the cell cycle, DNA repair, and oxidative phosphorylation were enriched in the mut‐high group.

**Conclusions:**

The prognosis of SCLC patients treated with chemotherapy was better among patients with more mutations in the FA metabolism pathway, and the underlying mechanisms could be found at the genome and transcriptome levels.

## Graphical abstract

We prospectively enrolled 52 extensive‐stage SCLC patients before treatment from local hospital to identify mutations related to patient prognosis. We found that the patients with high mutations (mut‐high) in the fatty acid (FA) metabolism pathway had better prognosis in the local hospital cohort and the Jiang's cohort than patients with low mutations (mut‐low). Then it was verified in George's cohort that mut‐high status was associated with a longer OS than mut‐low status. Finally, the possible mechanisms were explored from the genome and transcriptome levels.

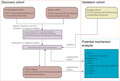

## 1 INTRODUCTION

Lung cancer is the most commonly diagnosed cancer worldwide and the most common cause of tumor‐related mortality.^1^, ^2^ Small‐cell lung cancer (SCLC), a unique type of lung cancer, accounts for approximately 13%–15% of lung cancers,^3^ and it was estimated that there were 29,654 new SCLC cases in the United States in 2017. This unique type is characterized by a higher growth rate and a higher degree of malignancy and can develop into a systemic disease earlier than other lung cancers.^4^, ^5^ For early limited‐stage small‐cell lung cancer (LS‐SCLC), surgery is the most common treatment option. However, this only applies to a very small number of patients who have not yet developed mediastinal lymph node metastasis, accounting for approximately 2%–5% of SCLC patients, and most patients need systemic management. For the remaining patients with extensive‐stage SCLC (ES‐SCLC), four cycles of chemotherapy with cisplatin combined with etoposide (the EP regimen) is the most widely used approach in clinical practice, with an overall response rate (ORR) of 50%–80%.^6^ Although most patients with SCLC show a good response in the early stages of treatment, the median overall survival (OS) time of ES‐SCLC is only 9 months, and only 2% of patients survive more than 5 years.^7^, ^8^ More than 90% of patients will develop drug resistance and eventually die from tumor recurrence.^4^, ^5^ A large number of existing studies suggest that the expression or mutation of certain genes may be related to the primary or secondary chemotherapy resistance of patients. Therefore, there is an urgent need to screen out people who can benefit from chemotherapy through effective indicators and to identify patients who are unlikely to benefit from chemotherapy for more appropriate treatment.

Next‐generation sequencing of tumor and adjacent normal tissues can reveal the genetic changes that have occurred in a tumor. Existing studies have suggested that alterations in key genes, such as tumor suppressor genes, may drive tumor progression and affect tumor cell responses to chemotherapeutics or targeted drugs, and many of these findings have been confirmed in clinical trials. For example, BCR‐ABL rearrangement mutations are related to the efficacy of small molecule inhibitors of the BCR‐ABL kinase, which leads to the wide usage of imatinib in the treatment of chronic myeloid leukemia.^9^ Oncogenic BRAF mutations are associated with the efficacy of BRAF inhibitors, and vemurafenib (BRAF V600E) has been demonstrated to extend the survival in clinical trials of patients with BRAF‐mutated skin melanoma.^10^ In addition, recent studies have suggested that some new genetic mutations may also be related to chemotherapeutic drug sensitivity and patient prognosis, but these claims need to be confirmed in further clinical trials.^11^, ^12^ There is much valuable genomics information stored in many public databases, including the The Cancer Genome Atlas (TCGA, https://portal.gdc.cancer.gov/), Gene Expression Omnibus (GEO, https://www.ncbi.nlm.nih.gov/geo/), and Genomics of Drug Sensitivity in Cancer (GDSC, https://www.cancerrxgene.org/) databases. In addition, many researchers have disclosed their sequencing data and clinical cohort data when their studies were publicly published. All of these resources provide opportunities for molecular medicine research.

A total of 31,117 pathway gene sets are stored in the Molecular Signatures Database (MSigDB, https://www.gsea‐msigdb.org/gsea/msigdb/), including 9 major collections, such as curated gene sets (C2), ontology gene sets (C5), and hallmark gene sets (H).^13^ Based on the gene sets provided in the MSigDB, gene set enrichment analysis (GSEA) and single sample GSEA (ssGSEA) can be applied to calculate the expression score of a specific pathway from an expression matrix, thereby inferring the expression of the pathway.^14^, ^15^ For instance, Zeng et al. calculated the expression score of each gene set in tumor tissues and then explored the expression of gene sets for predicting the prognosis of urothelial cancer patients treated with immune checkpoint inhibitors.^16^ In addition, many other studies used the data set provided by MSigDB to explore the differences in expression or mutations in a specific gene set between groups as potential clues to phenotypic differences between groups.^17^, ^18^, ^19^

Metabolic reprogramming is considered to be one of the 10 major characteristics of tumors.^20^ Warburg first observed that even under aerobic conditions, cancer cells mainly rely on glycolysis, which is called “aerobic glycolysis” or the “Warburg effect,” and increasing glycolysis allows glycolysis intermediates to enter various biosynthetic pathways, such as pathways that produce nucleosides and amino acids.^20^ In addition, alterations in fatty acid (FA) metabolism are often observed in rapidly proliferating tumor cells.^21^ FAs are composed of hydrocarbon chains and terminal carboxylic acid groups. Most of the time, they have an even number of carbon atoms, which can be saturated or unsaturated. They are necessary for energy storage, membrane proliferation, and signaling molecule generation.^21^ Normal cells tend to use exogenous substances, while tumor cells prefer de novo FA synthesis and show a higher level of FA synthesis.^22^ The increased FA de novo synthesis from acetyl‐CoA and NADPH enables the synthesis of more cell membranes and signaling molecules for rapidly proliferating and dividing tumor cells.^23^

Therefore, we prospectively collected tumor tissue samples from patients with ES‐SCLC before they received EP regimen treatment in Zhujiang Hospital of Southern Medical University to perform whole exome sequencing (WES) and combined these data with clinical follow‐up data to explore the relationship between the mutation frequency of the FA metabolism pathway and the prognosis of patients. Then, we obtained the published data sets for Jiang's cohort and George's cohort for verification. Finally, the RNA sequencing (RNA‐Seq) data from Jiang's cohort and the Genomics of Drug Sensitivity in Cancer (GDSC) database were used for subsequent mechanistic exploration.

## 2 METHODS

### 2.1 Workflow of the whole study

**FIGURE 1:**
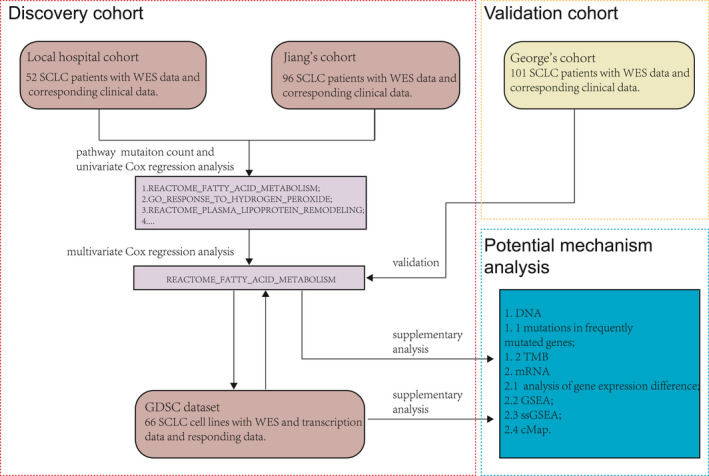
The whole workflow of this study. A three‐step method was adopted in this study: an initial mutational biomarker discovery step with the local hospital cohort and Jiang's cohort, a verification step with the independent George's cohort, and an investigation step for exploring the potential mechanisms at the genome and transcriptome levels

Figure 1 shows an overall flow chart of our research. We adopted a three‐step method: The first step was the initial mutational biomarker discovery step, where we systematically analyzed mutation data and clinical data from SCLC patients in the local cohort and Jiang's cohort to identify an independent mutational biomarker to predict the prognosis of patients treated with chemotherapy. In the second step, an independent cohort from a study by George et al. was used for verification. Finally, the data of cell lines from the GDSC database and clinical sample data from Jiang's cohort were used to explore potential mechanisms.

### 2.2 Collection and sequencing of samples from the local hospital cohort

Fresh‐frozen SCLC samples (NCT03162705, details of protocol available at https://www.clinicaltrials.gov/) were collected from April 2017 to June 2020 from 52 patients before treatment; peripheral blood was collected during follow‐up. This study was approved by the Ethics Committee of Southern Medical University. Whole exome sequencing (WES) was performed on the 52 fresh‐frozen SCLC samples, and WES was performed as mentioned in our previous research.^11^ Fifty‐two patients who met each of the inclusion criteria were included, and the detailed inclusion criteria were as follows: (1) age between 18 and 75 years old; (2) patients who had not undergone any treatment and were diagnosed with extensive SCLC by immunohistochemistry (IHC); (3) ECOG (Eastern Cooperative Oncology Group, ECOG) PS (performance status, PS) Scale score of 0 or 1; (4) expect to survive for at least 3 months; (5) peripheral routine blood tests and liver and kidney function met the following criterion (blood draw 7 days before the treatment began): white blood cell (WBC) count greater than 3.0 × 10^9/L or neutrophils (ANC) count more than 1.5 × 10^9/L; and hemoglobin (HGB) level greater than 80 g/L. Platelet (PLT) is greater than 100 × 10^9/L. Aspartate aminotransferase/alanine aminotransferase (AST/ALT) <3.0 times of normal range. Total bilirubin (TBIL) <1.5 times of normal range. Creatinine (CREAT) <1.5 times of normal range. (6) Female or male patients must adopt effective birth control measures, and (7) informed consent forms must be provided.

### 2.3 WES, transcription, clinical, and drug response data

For Jiang's cohort, the WES data and clinical records of 96 SCLC patients were obtained from the supplementary files provided by Jiang et al.,^24^ and standardized RNA‐Seq data for 49 patients were obtained from GSE60052 (http://www.ncbi.nlm.nih.gov/geo/query/acc.cgi?acc=GSE60052). For George's cohort, the WES data and clinical data of 101 SCLC patients were obtained from the supplementary files provided by George et al.^25^ In addition, we downloaded WES data and corresponding transcriptome data and drug responsiveness data for SCLC cell lines from the GDSC database (https://www.cancerrxgene.org/).

### 2.4 Gene sets related to the prognosis of SCLC patients treated with chemotherapy

Fifty hallmark gene sets (H), 5529 curated gene sets (C2), and 10192 ontology gene sets (C5) were downloaded from MSigDB (https://www.gsea‐msigdb.org/gsea/downloads.jsp). Based on the median mutation frequency of a certain gene set in the cohort, the samples were divided into a mut‐high group (excluding the median) and a mut‐low group for a certain pathway, and then univariate Cox proportional hazards survival analysis was used to test whether there was a significant difference in survival time (progression‐free survival [PFS] or OS) between groups. Multivariate proportional hazards survival analyses of the significant pathways and available clinical variables were applied to eliminate the interference of potential confounding factors, and then a reliable gene set representing a specific pathway related to the prognosis of SCLC patients treated with chemotherapy was chosen for subsequent analysis.

### 2.5 Differential gene expression (DGE) analysis and GSEA between the mut‐high and mut‐low groups

The mutation count of the FA metabolic pathway was related to the prognosis of SCLC patients, so the samples in cohorts were grouped based on the number of mutations in the FA metabolic pathway. In detail, if the number of mutations in the FA pathway gene set of a sample was greater than the median level, it was classified as mut‐high; otherwise, the sample was classified as mut‐low. The Limma R package was used to perform DGE analysis of the standardized transcription data.^26^ Then, the clusterProfiler R package was used to perform GSEA based on the gene set sorted by log fold change (FC) with 1000 iterations.^27^ The gene set variation analysis (GSVA) R package was used to evaluate the expression of gene sets in the sample, which reflects the expression level of the corresponding pathway.^15^ The limma R package was used again for differential pathway expression analysis. Gene sets C2, C5, and H were included in the GSEA and GSVA.

### 2.6 Connectivity Map (CMap) and Mechanism of Action (MoA) analysis

The SCLC cell lines in the GDSC database were grouped based on the mutation count in the FA metabolism pathway, and the half‐maximal inhibitory concentration (IC50) difference between the two groups was explored. The CMap biomedical software developed by the Broad Institute utilizes transcriptional expression data to explore relationships between genes, diseases, and therapeutics (https://clue.io/cmap).^28^, ^29^ In our study, to identify the potential compounds that reduce patient risk, CMap analysis was applied to compute enrichment scores through a web interface (https://portals.broadinstitute.org/cmap/), and build 02 was built as an Affymetrix‐based CMap data set. To query CMap, the genes were converted to probes in the GPL96 platform to be the exact queries, and CMap software was applied to compute an enrichment score that ranged from −1 to 1 and the corresponding permutation *p* value instantly. To further understand the drug MoA, all MoA and target information were downloaded from the same website.

### 2.7 Statistical analysis

The differences in clinical data, gene expression, pathway enrichment scores, log‐transformed IC50s (lnIC50s), and tumor mutational burden (TMB) between the mut‐high group and the mut‐low group were assessed, and the Wilcoxon test was used for the continuous variables. The survival and survminer R packages were used for Kaplan–Meier plots. The ComplexHeatmap Bioconductor package was used to visualize the top 20 mutated genes and genes with mutation frequencies of >5% in the FA metabolism pathway, and the Fisher's exact test was used to test whether the differences in mutation frequency between the two groups were significant. All the above analyses and visualizations were achieved in R (version 3.6.1) and RStudio (Version 1.2.1335). *p* < 0.05 was considered statistically significant, and all statistical tests were two‐sided.

## 3 RESULTS

### 3.1 Basic characteristics of the local hospital cohort and Jiang's cohort

From April 2017 to June 2020, a total of 52 patients diagnosed with ES‐SCLC with available biopsy tissues were enrolled in the local hospital cohort (clinical trial number: NCT03162705). The median age of patients was 63 years old. There were 46 male patients and 6 female patients in this cohort, and all of the patients received the EP regimen after sampling (Table 1). In Jiang's cohort, there were 96 patients whose data included both follow‐up data and tumor WES data. The median age of the patients was 57 (range, 36–77) years old. Eighty‐three (83/96, 86.5%) patients did not receive chemotherapy before sampling, and all patients received at least one chemotherapy cycle after sampling. In the latter cohort, the majority of patients were in tumor‐node‐metastasis (TNM) stage III, and the numbers of patients in stages I, II, III, and IV were 15, 16, 62, and 3, respectively. The numbers of smokers and nonsmokers were 71 and 25, respectively. The numbers of men and women were 83 and 13, respectively (Table 2).

**TABLE 1:** Baseline clinical characteristics of patients from the local cohort

Characteristics	Overall	mut‐low	mut‐high	*p* value
N	52	35	17	
Age (mean [SD])	62.96 (7.21)	63.03 (8.15)	62.82 (4.94)	0.924
Gender (%)				
Female	6 (11.5)	4 (11.4)	2 (11.8)	1
Male	46 (88.5)	31 (88.6)	15 (88.2)	
Smoke (%)				
Non‐smoker	9 (17.3)	9 (25.7)	0 (0.0)	0.055
Smoker	23 (44.2)	14 (40.0)	9 (52.9)	
MISS	20 (38.5)	12 (34.3)	8 (47.1)	
TNM stage (%)				
III	9 (17.3)	6 (17.1)	3 (17.6)	0.797
IV	22 (42.3)	16 (45.7)	6 (35.3)	
III/IV	21 (40.4)	13 (37.1)	8 (47.1)	
Chemotherapy exposed biopsy (%)				
Naive	52 (100.0)	35 (100.0)	17 (100.0)	—

**TABLE 2:** Baseline clinical characteristics of patients from the Jiang's cohort

Characteristics	Overall	mut‐low	mut‐high	*p* value
N	96	53	43	0.654
Age (mean [SD])	57.66 (8.33)	58.01 (8.56)	57.24 (8.11)	
Gender (%)				
Female	13 (13.5)	7 (13.2)	6 (14.0)	1
Male	83 (86.5)	46 (86.8)	37 (86.0)	
Smoke (%)				
Non‐smoker	25 (26.0)	12 (22.6)	13 (30.2)	0.543
Smoker	71 (74.0)	41(77.4)	30 (69.8)	
TNM stage (%)				
I	15 (15.6)	9 (17.0)	6 (14.0)	0.866
II	16 (16.7)	9 (17.0)	7 (16.3)	
III	62 (64.6)	34 (64.2)	28 (65.1)	
IV	3 (3.1)	1 (1.9)	2 (4.7)	
Chemotherapy exposed biopsy (%)				
Naive	83 (86.5)	47 (88.7)	36 (83.7)	0.685
Chemotherapy treated	13 (13.5)	6 (11.3)	7 (16.3)	

### 3.2 FA pathway mutations were associated with the prognosis of SCLC patients receiving chemotherapy

**FIGURE 2:**
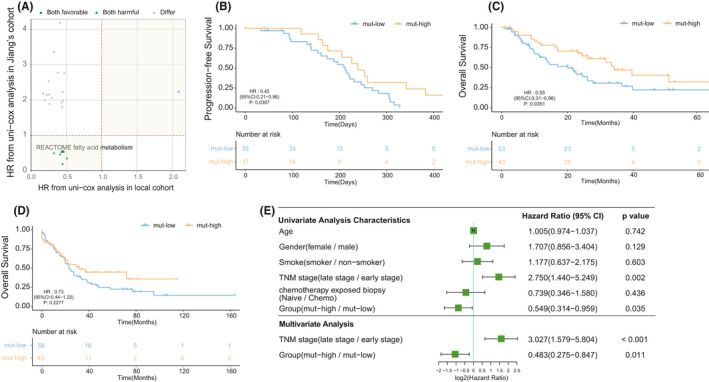
Fatty acid metabolism‐related mutations were associated with the prognosis of SCLC patients receiving chemotherapy. (A) Patients were grouped based on the median frequency of pathway mutations in their corresponding cohort, and the scatter plot shows the HRs of different pathways from the local hospital cohort and Jiang's cohort. All the points in the plot represent a pathway with *p* < 0.05 in the univariate Cox regression, and the green, blue, and gray dots indicate the pathways that were associated with better, worse, and different patient prognoses in the two cohorts, respectively. (B) Kaplan–Meier survival curves of the PFS of the local hospital cohort comparing patients in the mut‐high group and the mut‐high group. (C) Kaplan–Meier survival curves of the OS of Jiang's cohort comparing patients in the mut‐high group and the mut‐high group. (D) Kaplan–Meier survival curves of the OS of the George's cohort comparing patients in the mut‐high group and the mut‐high group. (E) Univariate and multivariate analyses in Jiang's cohort. (A–D) Differences between the mut‐high and mut‐high groups were assessed using univariate Cox analysis

Gene sets representing different pathways were downloaded from MSigDB (https://www.gsea‐msigdb.org/gsea/downloads.jsp), and the mutation frequency of each pathway in the sample was calculated. In the discovery stage, both the local hospital cohort and Jiang's cohort contained only patients from China. We calculated the mutation frequency of each sample in the two cohorts based on the genes in the C2, C5, and H gene sets. We performed univariate Cox analysis of patients stratified by the median value of mutational counts in each pathway. In these two cohorts, we compared the HR of pathways that was significantly related to the prognosis and found that a total of 10 gene sets had consistent patterns in both cohorts (Figure 2A): one highly mutated pathway was related to a worse prognosis and nine highly mutated pathways were associated with a better prognosis. The prognosis of patients with high mutational counts (mut‐high) in the FA metabolism pathway was significantly better than that of patients with low mutational counts (mut‐low) in this pathway. Specifically, in the local hospital cohort, patients with a high number of mutations (patients with more than one mutation) in the FA metabolism pathway had a longer PFS time than those in the mut‐low group (median PFS [95% CI]: 7.07 [5.36 – 8.43] months vs. 7.97 [6.83‐not reached] months; HR [95% CI]: 0.446 [0.207–0.959]; *p* = 0.0387](Figure 2B). In Jiang's cohort, the mut‐high group which included patients with more than two mutations in the FA metabolism pathway had a longer OS time than the mut‐low group (median OS [95% CI]: 20.1 [13.3–27.8] months vs. 34.2 [26.1‐not reached] months; HR [95% CI]: 0.549 [0.314–0.960]; *p* = 0.0351) (Figure 2C).

Then, we compared the clinical characteristics of patients in the local cohort and Jiang's cohort. There was no significant difference between the mut‐high and mut‐low groups in either cohort (Table 1 and Table 2). In addition, we included the available clinical variables and performed univariate and multivariate Cox regression analyses that took the patients’ age, sex, tumor stage, and previous chemotherapy into account to exclude confounding factors. In Jiang's cohort, because there were only three patients with stage IV disease, we combined stage III and stage IV patients into an advanced SCLC group and combined the remaining stage I and stage II patients into an early SCLC group. In the univariate analysis (Figure 2C,E), we found that in addition to a high number of mutations in FA metabolism pathways being associated with longer OS time (HR = 0.549, 95% CI, 0.314–0.959, *p* = 0.035), late SCLC was associated with a shorter OS time (HR = 2.750, 95% CI 1.440–5.249, *p* = 0.002), which was consistent with the actual clinical observations. In the multivariate analysis that included the significant variables from the univariate analysis, tumor stage and the mutation levels of the FA metabolism pathway were still related to the prognosis (HR = 3.027, 95% CI 1.579–5.804, *p* < 0.001; HR = 0.483, 95% CI 0.275–0.847, *p* = 0.011, respectively).

In the validation phase, we again verified that mut‐high status (patients with more than one mutation) of the FA metabolism pathway was associated with a better prognosis than mut‐low status(median OS [95% CI]: 23 (20‐34) months vs. 32 [18‐not reached] months; HR[95%CI]: 0.73 [0.44–1.22]; *p* = 0.2277) in George's cohort(Figure 2D), where most of the patients were Caucasian.

### 3.3 Differences in gene mutation profiles between the mut‐high and mut‐low groups

**FIGURE 3:**
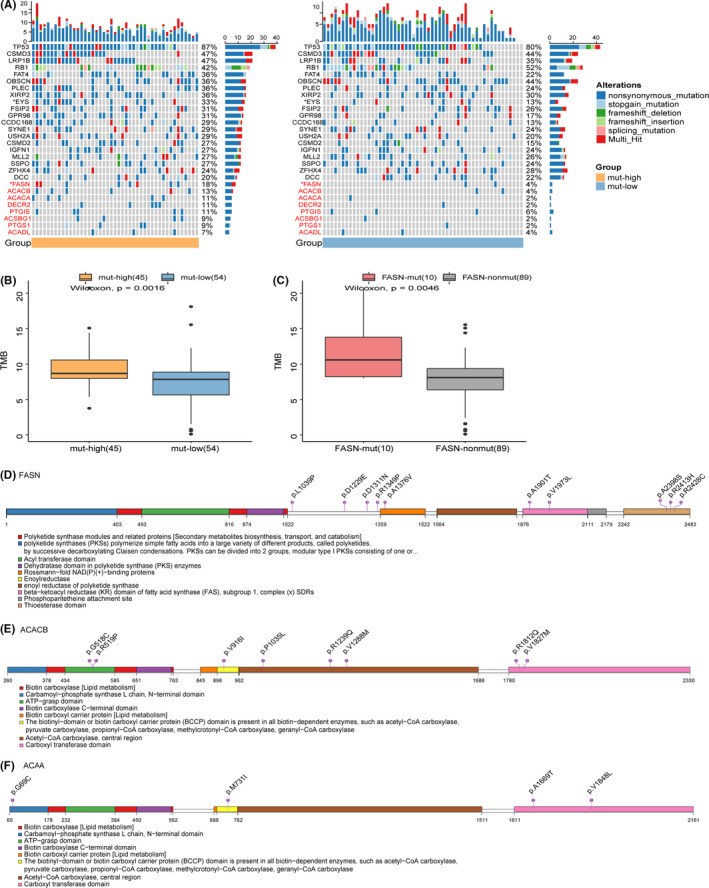
The landscape of genomic alterations and lollipop charts of FASN mutation sites in Jiang's cohort. (A) Mutation profile of the top 20 currently mutated genes and genes in FA metabolism with overall mutation frequency >5% in 99 SCLC samples from Jiang's cohort, grouped by the frequency of mutations in the FA metabolism pathway. The gene symbols are marked on the left side: the symbols of genes in black font color are the top 20 mutated genes, and the symbols of genes in red font color are genes in the FA metabolism pathway. Asterisks indicate the relationship between the mut‐high/mut‐low groups, and those mutated genes were tested using the Fisher's exact test. ****p < 0.0001, ***p < 0.001, **p < 0.01, *p < 0.05. (B) The TMB in the mut‐high group was significantly higher than that in the mut‐low group. The values of the TMB are plotted on a log scale. (C) The TMB of the patients with mutated FASN was significantly higher than that of patients without mutated FASN. The values for TMB are plotted on a log scale. (D–F) Amino acid positions of FASN, ACACB, and ACACA mutations. Numbers in circles represent the frequency of the corresponding site mutation

We grouped the patients in Jiang's cohort based on counts of mutations in the FA metabolism pathway and summarized the clinical characteristics of each group (Table 1). We found that there was no significant difference in clinical characteristics between the two groups, so the data sets were balanced. Figure 3A shows the top 20 mutated genes and the genes in FA metabolism pathways with an overall mutation frequency greater than 5%. TP53 was the most frequently mutated gene, whose mutation frequencies in the two groups were 87% and 80%, respectively. CSMD3 (47% vs. 44%), LRP1B (47% vs. 35%), RB1 (42% vs. 52%), and OBSCN (36% vs. 44%) were also commonly mutated. Among the top 20 mutated genes, the mutation frequency of EYS in the mut‐high group was significantly higher than that in the mut‐low group (33% vs. 13%). For genes in the FA metabolism pathway, the mutation frequencies of fatty acid synthase (FASN), ACACB, ACACA, DECR2, and PTGIS were greater than 10% in the mut‐high group. Among these genes, FASN was the most commonly mutated gene and the only gene whose mutation frequency in the mut‐high group was significantly higher than that in the mut‐low group (18% vs. 4%). We applied the same analysis in the local hospital cohort and found that the mutation frequency of FASN was significantly higher in the mut‐high group than in the mut‐low group (12% vs. 0%).

In addition, we calculated the TMB for each sample in each cohort. The TMB in the mut‐high group was significantly higher than that in the mut‐low group in Jiang's cohort (*p* = 0.0016) (Figure 3B). Considering that the mutation frequency of FASN was significantly different between the two groups, we explored the association between FASN mutations and TMB. As expected, we found that the TMB in the group with mutated FASN was significantly higher than that in the group without mutated FASN (Figure 3C). Genomic instability is one of the hallmarks of tumors, so we compared the mutations in the genes related to DNA damage and repair pathways (DDR) between the two groups. However, there was no significant difference between the two groups (data not shown). This result suggests that there may be other mechanisms that lead to differences in patient prognosis between the two groups.

Next, we visualized the mutations of genes with high mutation frequency in the FA metabolism pathway (Figure 3D). FASN is a complex gene containing multiple enzyme domains, and the mutations were mainly located in the region that lacks catalytic activity, including p.L1039P, p.D1229E, p.D1311N, and p.R1349P. Some other mutations were located in the thioesterase domain, including 3p.A2398S, 1p.R2413H, and 1p.R2428C, which may cause loss of function. Other key genes in FA metabolism, including ACACB, ACACA, DECR2, and PTGIS, had recurrent mutations. Mutations in the above genes mainly occurred in the active sites (Figure 3E–F).

### 3.4 Differences in gene and pathway expression between the mut‐high and mut‐low groups

**FIGURE 4:**
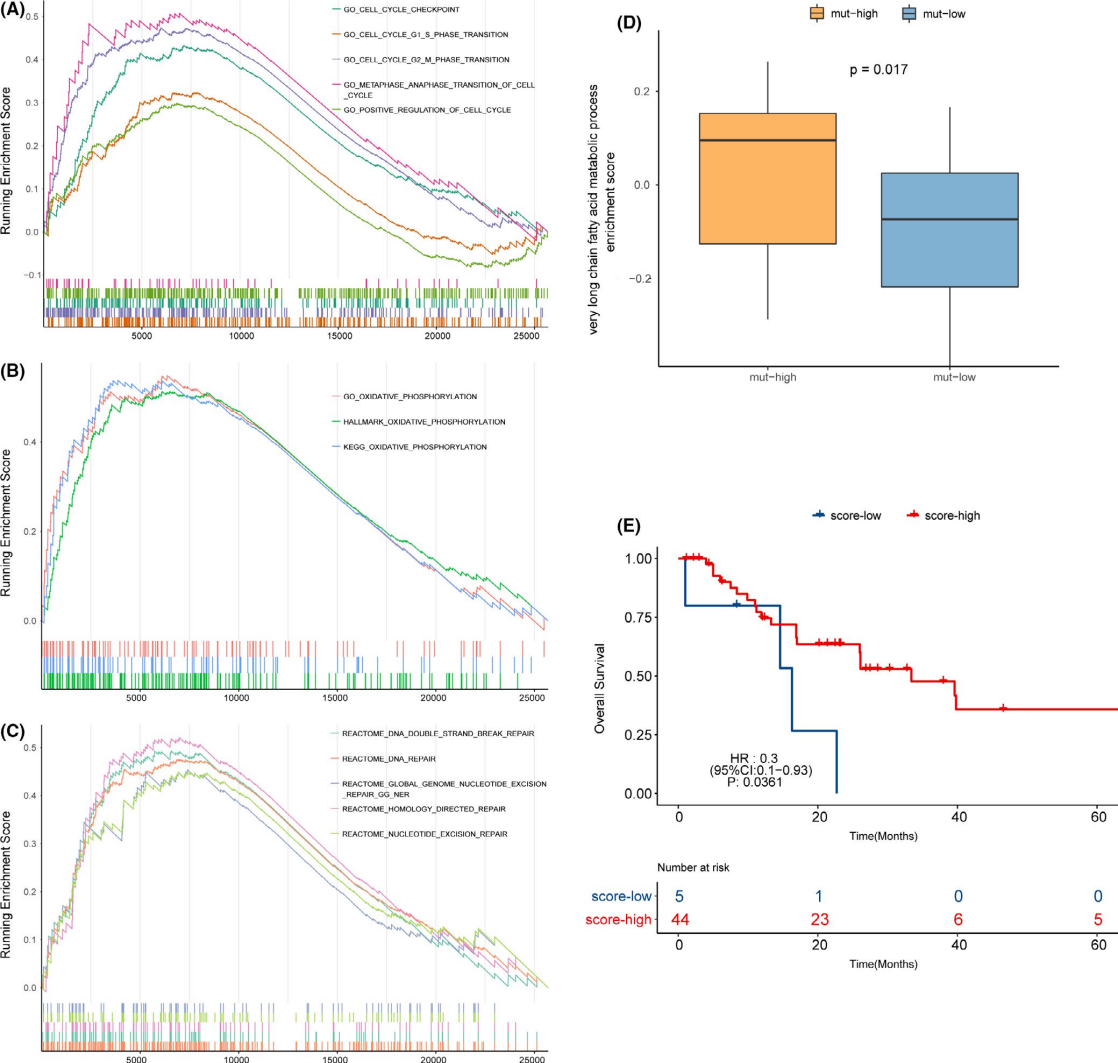
Representative differentially enriched pathways between the mut‐high and mut‐low groups and the pathway related to patient survival. (A–C) Pathways related to the cell cycle, DNA damage and repair, and oxidative phosphorylation were enriched in the mut‐high group compared with the mut‐low group. (D) The very long FA metabolic process pathway scored higher in the mut‐high group than in the mut‐low group. (E) A higher score for the very long FA metabolic process pathway was associated with a better prognosis (HR: 0.3, 95% CI: 0.1−0.93). FA, fatty acid

We analyzed the transcription data of 49 patients with both WES data and prognostic information to further explore potential mechanisms. First, when the mut‐high and mut‐low groups were compared, there were a total of 50 upregulated genes and 77 downregulated genes in the mut‐high group (*p* value <0.05 and FC >2/1 or FC <1/2). Then, we performed GSEA to identify the relationships between upregulated or downregulated genes. Pathways related to the cell cycle, DNA damage and repair, and oxidative phosphorylation were enriched in the mut‐high group (Figure 4A–C). In contrast, some pathways related to lipid localization and binding were mainly enriched in the mut‐low group. Pathways related to arachidonic acid metabolism and bile salt circulation were also enriched in the mut‐low group.

### 3.5 Relationship between mutations in FA metabolism pathways, pathway expression, and patient prognosis

Previous results suggested that mutations in FA metabolism pathways do not directly affect the patient prognosis by affecting the metabolism of FAs or other substances. Therefore, we further explored which pathway mutations are related not only to FA pathway mutations but also to patient prognosis to identify the potential mechanism that may affect patient outcomes. We found that the very long fatty acid metabolic process pathway scored higher in the mut‐high group (Figure 4D, t = 2.3, *p* = 0.026), and a higher score of this pathway was associated with a better prognosis (Figure 4E). In the univariate Cox analysis of the very long FA metabolic process pathway, patients with higher scores were at a lower risk than those with lower scores (median OS (95% CI): 16.3 (14.6‐not reached) months vs. 33.4 (17.0‐not reached) months; HR (95% CI): 0.302 (0.098–0.925); *p* = 0.036). In the following multivariate Cox regression, when the influence of clinical factors such as tumor stage, sex, age, and smoking was excluded, this pathway also showed a trend to be prognostic (HR = 0.315, 95% CI, 0.098–1.018, *p* = 0.054).

### 3.6 Mutations in the FA metabolism pathway affect the responsiveness of SCLC cell lines to some drugs

**FIGURE 5:**
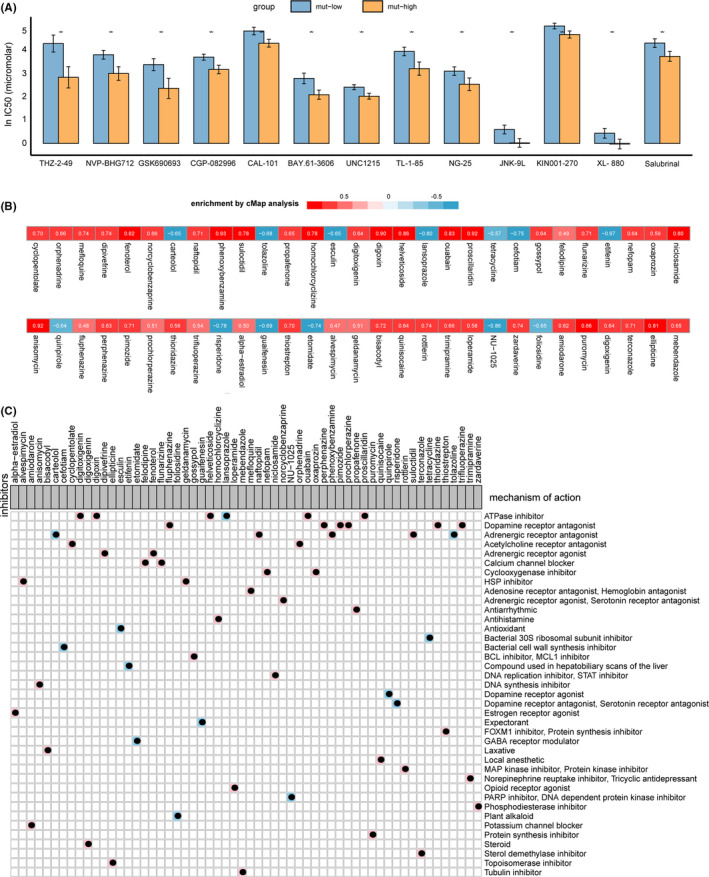
Comparison of drug responsiveness between the mut‐high and mut‐low groups and the results of CMap analysis. (A) LnIC50 values of drugs between the mut‐high group and the mut‐low group. The IC50s reported in the GDSC database were log transformed. The differences in the lnIC50 values of different drugs between the mut‐high and mut‐low cell lines were tested using the Mann–Whitney *U* test. ns: not significant; *p < 0.05; **p < 0.01; ***p < 0.001. (B) Heatmap showing the enrichment score of each drug from the CMap analysis for cell lines in the GDSC database. Blue corresponds to a negative enrichment score; red corresponds to a positive score. (C) Heatmap showing the drugs from the CMap analysis that share an MoA. The background colors (blue and pink) correspond to negative and positive enrichment scores, respectively

Next, we explored the correlation between the frequency of mutations in FA metabolism pathways and the responsiveness of SCLC cell lines to compounds in the GDSC database. We found that only the mut‐low group demonstrated significantly higher lnIC50s than the mut‐high group (Figure 5A). These drugs included THZ‐2–49, CGP‐082996, and KIN001‐270, which target the cell cycle, as well as NVP‐BHG712, GSK690693, CAL‐101, BAY.61–3606, UNC1215, TL‐1–85, NG‐25, JNK‐9L, XL‐880, and salubrinal.

### 3.7 CMap analysis revealed that ATPase inhibitors could reduce patient risk

CMap is a gene expression profile database based on interventional gene expression developed by the Broad Institute, which can be used to reveal the functional connections between small molecule compounds, genes, and diseases. Therefore, we used the data of SCLC cell lines to explore potential drugs that could reduce patient risk (Figure 5B,C). Among the four drugs with scores >0.9 and *p* < 0.05, there were two ATPase inhibitors (2/4), an adrenergic receptor antagonist (1/4), and a DNA synthesis inhibitor. Among the 11 drugs with scores >0.8 and *p* < 0.05, 4 were ATPase inhibitors, 2 were adrenergic antagonists, 1 was a DNA synthesis inhibitor, 1 was a protein synthesis inhibitor, 1 was a topoisomerase inhibitor, 1 was a DNA replication inhibitor, and 1 was a STAT inhibitor. Among the drugs with the opposite trend, only one drug—a PARP inhibitor, NU‐1025—satisfied the score <−0.8 and *p* < 0.05 criteria.

## 4 DISCUSSION

In our research, we found that the mut‐high group had a better prognosis than the mut‐low group. Analysis of the underlying mechanisms suggested that there were mutation differences in key genes in FA metabolism between the two groups, and some pathway components related to the cell cycle, DNA damage and repair, and oxidative phosphorylation were differentially expressed. Finally, the results based on CMap analysis suggest that ATPase inhibitors may reduce patient risk.

Genomic instability is a characteristic of tumors, manifested by the accumulation of mutational burden. When comparing the TMB of the mut‐high group and the mut‐low group, we found that the TMB of the mut‐high group was significantly higher. This may be because the mut‐high group itself included tumor samples with a higher mutational background. However, the classification of mut‐high and mut‐low groups in this study was based on the median mutation counts in the FA metabolism pathway. Specifically, the medians in Jiang's and local hospital cohorts were 2 and 1, respectively, suggesting that the grouping may not be the cause of the difference in TMB between the two groups. Previous studies have suggested that the accumulation of mutations in the DDR pathway leads to higher TMB because more unrepaired mutations will accumulate in the cells.^30^ However, we did not find any difference in the mutation loads of the overall DDR pathway or each DDR sub‐pathway between the two groups, which may indicate that there were other reasons for the difference in TMB between the two groups.

FASN is a key regulatory molecule in lipid metabolism and plays an important role in the growth and survival of tumor cells with lipogenic phenotypes.^31^ In our study, the mutational frequency of FASN was significantly higher in the mut‐high group (18% vs. 4%). FASN is a large multienzyme complex whose monomer protein is approximately 270 kDa. It contains six independent enzyme grooves, which can work together to generate 16‐carbon chain saturated FAs from acetyl‐CoA (CoA) and malonyl‐CoA (FA) in the presence of nicotinamide adenine dinucleotide hydrogen phosphate (NADPH). Although a single FASN molecule contains all the crucial enzymes for palmitate synthesis, the dimerization of FASN is important for its function.^32^ Approximately 1/4 of the length of FASN is located in the middle range, which lacks catalytic activity but is necessary for the formation of dimers. The mutations in FASN, including p.L1039P, p.D1229E, p.D1311N, and p.R1349P, were mainly located in the crucial middle part, which may affect dimer formation and thus affect its function. In addition, thioesterase is the key enzyme that controls chain length during FA synthesis,^33^ and some mutations were located in the thioesterase domain of FASN, including 3p.A2398S, 1p.R2413H, and 1p.R2428C, which may also cause functional deficiency. Next, we also reviewed recurrent gene mutations in the FA metabolism pathway. The affected genes were key genes in FA metabolism, including ACACB, ACACA, DECR2, and PTGIS, with mutations mainly in the active sites. Although there was no significant regional enrichment, the role of these mutations in the process of tumorigenesis should not be ignored.

Both cisplatin and etoposide are cell cycle‐specific drugs, and both can form cross‐links with cell DNA to affect DNA function,^34^ thereby inducing cell death.^35^ The GSEA results suggested that pathways related to the cell cycle and DNA damage and repair were enriched in the mut‐high group, which may be a potential mechanism for the difference in prognosis between the mut‐high group and the mut‐low group. In addition, pathways related to oxidative phosphorylation, the tricarboxylic acid cycle, and respiratory electron transfer processes were also enriched in the mut‐high group. In contrast, some pathways related to lipid localization and binding were mainly enriched in the mut‐low group. Arachidonic acid metabolism‐ and bile acid salt circulation‐related pathways were also enriched in the mut‐low group. This may indicate that normal oxidative metabolism was more enriched in the mut‐high group or that the degree of tumor metabolic reprogramming in the mut‐high group was lower than that of the mut‐low group.

Previous studies have suggested that very long‐chain FAs have a carbon chain length >= 22 and are beta‐oxidized in peroxisomes^36^; these FAs generate heat instead of ATP and ultimately disperse in cells.^37^ The pathway of very long‐chain FA metabolism scored significantly higher in the low‐risk group, and a higher score for this pathway was related to a better patient prognosis (HR = 0.302, 95% CI: 0.098–0.925, *p* = 0.036), which may be one of the possible mechanisms for the difference in survival between the two groups. In the subsequent multivariate analysis including clinical variables, the score of this pathway showed a trend to be prognostic (HR = 0.315, 95% CI: 0.098–1.018, *p* = 0.054), which was mainly due to the small sample size.

The current version of the GDSC database contains nearly 1000 tumor cell lines, covering common tumor types.^38^ It also contains 518 kinds of drugs, including cytotoxic drugs and targeted drugs. In our research, only the mut‐low group demonstrated a significantly higher lnIC50 than the mut‐high group for some drugs, suggesting that the mut‐low group may be less sensitive to cytotoxic chemotherapeutics than the mut‐high group on a global scale.

CMap is a gene expression profile database based on interventional gene expression developed by the Broad Institute, which can be used to reveal the functional connections between small molecule compounds, genes, and diseases.^28^, ^29^ The results of CMap analysis suggested that ATPase inhibitors may reduce patient risk, thereby improving the prognosis of patients in the high‐risk group. Specifically, the enriched ATPase inhibitors referred to Na^+^/K^+^‐ATPase inhibitors, including digitoxigenin, digoxin, helveticoside, ouabain, and proscillaridin, which are cardiac glycosides that have been studied in the contexts of inducing tumor apoptosis, inhibiting tumor cell growth, and inhibiting topoisomerase activity.^39^ A recent study showed that circulating tumor cells (CTCs) with stem cell‐like characteristics can methylate DNA to achieve metastatic seeding, while ouabain can rescue this process by increasing the intracellular Ca^2^+ concentration.^40^ The results of the mentioned studies suggest that ATPase inhibitors may play an antitumor role.

The advantage of this study is that it combines data from two publicly published cohorts to verify our conclusions from the local hospital cohort, making our conclusions more robust. In addition, we used bioinformatics analysis to explore the potential mechanisms at the genome and transcriptome levels. Finally, we explored the relationship between mutations in FA metabolism pathways and the IC50 of cytotoxic drugs and used CMap analysis to identify potential drugs that could reduce patient risk. However, there were also some limitations. First, we found that a high number of mutations in FA metabolism pathways may be associated with a better prognosis than a low number of mutations in SCLC patients treated with chemotherapy. However, there were too many missing values in treatment response records that made it impossible to compare the disease control rate between groups directly, and there may be other therapeutic factors aside from chemotherapy, such as thoracic radiotherapy, that affect the prognosis of patients in the three clinical cohorts. Second, we grouped samples based on the median counts of FA metabolism pathway mutations. The medians in the three clinical cohorts were slightly different (local hospital cohort, 1; Jiang's cohort, 2; and George's cohort, 1), and there was no uniform cutoff value. Finally, when we used the GDSC database to explore potential drugs that can reduce patient risk, we screened cardiac glycosides through strict conditions. Although these drugs have an antitumor effect in a variety of tumors, they have rarely been reported in SCLC, so our conclusion requires further validation.

## 5 CONCLUSION

In our study, we found that SCLC patients with a high number of mutations in the FA metabolism pathway had a better prognosis than patients with a low number of mutations after receiving the EP regimen. Mechanistic analysis suggested that these findings may be explained by the observations that the mut‐high group demonstrated a higher TMB and a higher number of FASN mutations than the mut‐low group. Analysis of expression data revealed that pathways related to the cell cycle, DNA damage and repair, and oxidative phosphorylation pathways were more enriched in the mut‐high group. In addition, the level of very long‐chain FA metabolism was higher in the mut‐high group, which may be a metabolic factor affecting the prognosis of patients. Analysis of these data and data from the GDSC database suggested that the mut‐low group tends to be more resistant to chemotherapeutics. The results of CMap analysis suggested that NA^+^/K^+^‐ATPase inhibitors could reduce patient risk, but this requires further verification.

## CONFLICT OF INTEREST

The authors declare no conflict of interest.

## ETHICS STATEMENT

Fresh‐frozen SCLC samples (NCT03162705, details of protocol available at https://www.clinicaltrials.gov/) were collected from April 2017 to June 2020 from 52 patients before treatment; peripheral blood was collected during follow‐up. This study was approved by the Ethics Committee of Southern Medical University (Guangzhou, China).

## Data Availability

The data of SCLC patients from the GSE60052 cohort and the George cohort were obtained from the supplementary files provided by their authors. The standardized RNA‐Seq data for 49 patients were obtained from GSE60052 (http://www.ncbi.nlm.nih.gov/geo/query/acc.cgi?acc=GSE60052). The data for SCLC cell lines were obtained from the GDSC database (https://www.cancerrxgene.org/). Addition, gene sets used in our study were downloaded from MSigDB (https://www.gsea‐msigdb.org/gsea/downloads.jsp).

## References

[1] Herbst RS , Morgensztern D , Boshoff C . The biology and management of non‐small cell lung cancer. Nature. 2018;553(7689):446‐454. 29364287 10.1038/nature25183

[2] Torre LA , Siegel RL , Ward EM , Jemal A . Global cancer incidence and mortality rates and trends–an update. Cancer Epidemiol Biomarkers Prev. 2016;25(1):16‐27. 26667886 10.1158/1055-9965.EPI-15-0578

[3] Sabari JK , Lok BH , Laird JH , Poirier JT , Rudin CM . Unravelling the biology of SCLC: implications for therapy. Nat Rev Clin Oncol. 2017;14(9):549‐561. 28534531 10.1038/nrclinonc.2017.71 PMC5843484

[4] Schiller JH , Adak S , Cella D , DeVore RR , Johnson DH . Topotecan versus observation after cisplatin plus etoposide in extensive‐stage small‐cell lung cancer: E7593–a phase III trial of the Eastern Cooperative Oncology Group. J Clin Oncol. 2001;19(8):2114‐2122. 11304763 10.1200/JCO.2001.19.8.2114

[5] Jett JR , Schild SE , Kesler KA , Kalemkerian GP . Treatment of small cell lung cancer: diagnosis and management of lung cancer, 3rd ed: American College of Chest Physicians evidence‐based clinical practice guidelines. Chest. 2013;143(5 Suppl):e400S‐e419S. 23649448 10.1378/chest.12-2363

[6] Rossi A , Di Maio M , Chiodini P , et al. Carboplatin‐ or cisplatin‐based chemotherapy in first‐line treatment of small‐cell lung cancer: the COCIS meta‐analysis of individual patient data. J Clin Oncol. 2012;30(14):1692‐1698. 22473169 10.1200/JCO.2011.40.4905

[7] Ogino H , Hanibuchi M , Kakiuchi S , et al. Analysis of the prognostic factors of extensive disease small‐cell lung cancer patients in Tokushima university hospital. J Med Invest. 2016;63(3–4):286‐293. 27644574 10.2152/jmi.63.286

[8] Chute JP , Chen T , Feigal E , Simon R , Johnson BE . Twenty years of phase III trials for patients with extensive‐stage small‐cell lung cancer: perceptible progress. J Clin Oncol. 1999;17(6):1794‐1801. 10561217 10.1200/JCO.1999.17.6.1794

[9] Druker BJ , Guilhot F , O'Brien SG , et al. Five‐year follow‐up of patients receiving imatinib for chronic myeloid leukemia. N Engl J Med. 2006;355(23):2408‐2417. 17151364 10.1056/NEJMoa062867

[10] Chapman PB , Hauschild A , Robert C , et al. Improved survival with vemurafenib in melanoma with BRAF V600E mutation. N Engl J Med. 2011;364(26):2507‐2516. 21639808 10.1056/NEJMoa1103782 PMC3549296

[11] Li M , Lin A , Luo P , et al. DNAH10 mutation correlates with cisplatin sensitivity and tumor mutation burden in small‐cell lung cancer. Aging. 2020;12(2):1285‐1303. 31959735 10.18632/aging.102683 PMC7053592

[12] Qiu Z , Lin A , Li K , et al. A novel mutation panel for predicting etoposide resistance in small‐cell lung cancer. Drug Des Devel Ther. 2019;13:2021‐2041. 10.2147/DDDT.S205633 PMC6594009 31417239

[13] Liberzon A , Birger C , Thorvaldsdóttir H , Ghandi M , Mesirov JP , Tamayo P . The Molecular Signatures Database (MSigDB) hallmark gene set collection. Cell Syst. 2015;1(6):417‐425. 26771021 10.1016/j.cels.2015.12.004 PMC4707969

[14] Subramanian A , Tamayo P , Mootha VK , et al. Gene set enrichment analysis: a knowledge‐based approach for interpreting genome‐wide expression profiles. Proc Natl Acad Sci. 2005;102(43):15545‐15550 16199517 10.1073/pnas.0506580102 PMC1239896

[15] Hänzelmann S , Castelo R , Guinney J . GSVA: gene set variation analysis for microarray and RNA‐seq data. BMC Bioinformatics. 2013;14:7. 23323831 10.1186/1471-2105-14-7 PMC3618321

[16] Zeng D , Ye Z , Wu J , et al. Macrophage correlates with immunophenotype and predicts anti‐PD‐L1 response of urothelial cancer. Theranostics. 2020;10(15):7002‐7014. 32550918 10.7150/thno.46176 PMC7295060

[17] Wang Y , Cai YY , Herold T , et al. An immune risk score predicts survival of patients with acute myeloid leukemia receiving chemotherapy. Clin Cancer Res. 2020;27(1):255–266. 33262139 10.1158/1078-0432.CCR-20-3417

[18] Owonikoko TK , Dwivedi B , Chen Z , et al. YAP1 expression in SCLC defines a distinct subtype with T‐cell‐inflamed phenotype. J Thorac Oncol. 2020;16(3):464–476. 33248321 10.1016/j.jtho.2020.11.006 PMC7920957

[19] Awasthi S , Berglund A , Abraham‐Miranda J , et al. Comparative genomics reveals distinct immune‐oncologic pathways in African American men with prostate cancer. Clin Cancer Res. 2020;27(1):320–329. 33037017 10.1158/1078-0432.CCR-20-2925 PMC8042600

[20] Hanahan D , Weinberg RA . Hallmarks of cancer: the next generation. Cell. 2011;144(5):646‐674. 21376230 10.1016/j.cell.2011.02.013

[21] Santos CR , Schulze A . Lipid metabolism in cancer. The FEBS J. 2012;279(15):2610‐2623. 22621751 10.1111/j.1742-4658.2012.08644.x

[22] Currie E , Schulze A , Zechner R , Walther TC , Farese RJ . Cellular fatty acid metabolism and cancer. Cell Metab. 2013;18(2):153‐161. 23791484 10.1016/j.cmet.2013.05.017 PMC3742569

[23] Röhrig F , Schulze A . The multifaceted roles of fatty acid synthesis in cancer. Nat Rev Cancer. 2016;16(11):732‐749. 27658529 10.1038/nrc.2016.89

[24] Jiang L , Huang J , Higgs BW , et al. Genomic landscape survey identifies SRSF1 as a key oncodriver in small cell lung cancer. Plos Genet. 2016;12(4):e1005895. 27093186 10.1371/journal.pgen.1005895 PMC4836692

[25] George J , Lim JS , Jang SJ , et al. Comprehensive genomic profiles of small cell lung cancer. Nature. 2015;524(7563):47‐53. 26168399 10.1038/nature14664 PMC4861069

[26] Ritchie ME , Phipson B , Wu DI , et al. limma powers differential expression analyses for RNA‐sequencing and microarray studies. Nucleic Acids Res. 2015;43(7):e47. 25605792 10.1093/nar/gkv007 PMC4402510

[27] Yu G , Wang LG , Han Y , He QY . clusterProfiler: an R package for comparing biological themes among gene clusters. OMICS. 2012;16(5):284‐287. 22455463 10.1089/omi.2011.0118 PMC3339379

[28] Subramanian A , Narayan R , Corsello SM , et al. A next generation connectivity map: L1000 platform and the first 1,000,000 profiles. Cell. 2017;171(6):1437‐1452. 29195078 10.1016/j.cell.2017.10.049 PMC5990023

[29] Lamb J , Crawford ED , Peck D , et al. The connectivity map: using gene‐expression signatures to connect small molecules, genes, and disease. Science. 2006;313(5795):1929‐1935. 17008526 10.1126/science.1132939

[30] Chalmers ZR , Connelly CF , Fabrizio D , et al. Analysis of 100,000 human cancer genomes reveals the landscape of tumor mutational burden. Genome Med. 2017;9(1):34. 28420421 10.1186/s13073-017-0424-2 PMC5395719

[31] Fhu CW , Ali A . Fatty acid synthase: an emerging target in cancer. Molecules. 2020;25(17). 10.3390/molecules25173935 PMC7504791 32872164

[32] Chirala SS , Wakil SJ . Structure and function of animal fatty acid synthase. Lipids. 2004;39(11):1045‐1053. 15726818 10.1007/s11745-004-1329-9

[33] Deng X , Chen L , Hei M , Liu T , Feng Y , Yang GY . Structure‐guided reshaping of the acyl binding pocket of ‘TesA thioesterase enhances octanoic acid production in E. coli. Metab Eng. 2020;61:24‐32. 32339761 10.1016/j.ymben.2020.04.010

[34] Rottenberg S , Disler C , Perego P . The rediscovery of platinum‐based cancer therapy. Nat Rev Cancer. 2020.21(1):37–50. 33128031 10.1038/s41568-020-00308-y

[35] van Maanen JM , Retèl J , de Vries J , Pinedo HM . Mechanism of action of antitumor drug etoposide: a review. J Natl Cancer Inst. 1988;80(19):1526‐1533. 2848132 10.1093/jnci/80.19.1526

[36] Carragher F , Champion M . Chapter 24 ‐ Inherited metabolic disease. In: Marshall WJ , Lapsley M , Day AP , Ayling RM , eds. Clinical Biochemistry: Metabolic and Clinical Aspects. 3rd ed. Churchill Livingstone; 2014:461‐483.

[37] Risé P , Paroni R , Petroni A . Chapter 3 ‐ Peroxisomal pathways, their role in neurodegenerative disorders and therapeutic strategies. In: Watson RR , De Meester F , eds. Omega‐3 Fatty Acids in Brain and Neurological Health. Academic Press; 2014:19‐30.

[38] Iorio F , Knijnenburg TA , Vis DJ , et al. A landscape of pharmacogenomic interactions in cancer. Cell. 2016;166(3):740‐754. 27397505 10.1016/j.cell.2016.06.017 PMC4967469

[39] Li R‐Z , Fan X‐X , Duan F‐G , et al. Proscillaridin A induces apoptosis and suppresses non‐small‐cell lung cancer tumor growth via calcium‐induced DR4 upregulation. Cell Death Dis. 2018;9(6):696. 29899551 10.1038/s41419-018-0733-4 PMC5999972

[40] Gkountela S , Castro‐Giner F , Szczerba BM , et al. Circulating tumor cell clustering shapes DNA methylation to enable metastasis seeding. Cell. 2019;176(1–2):98‐112. 30633912 10.1016/j.cell.2018.11.046 PMC6363966

